# Severe Invasive Pneumococcal Disease in Two Siblings: Austrian Syndrome and Infected Aortic Aneurysm

**DOI:** 10.3390/idr18050112

**Published:** 2026-10-04

**Authors:** Carolina V. Monteiro, Catarina Pestana Santos, Miguel Silva, Sandra Rebelo, António Martins, Lurdes Santos, Sofia R. Valdoleiros

**Affiliations:** 1 Department of Infectious Diseases, Hospital de São João, Unidade Local de Saúde de São João, 4200-319 Porto, Portugal; antonio.pedro.martins@ulssjoao.min-saude.pt (A.M.); maria.santos@ulssjoao.min-saude.pt (L.S.); sofiadevaldoleiros@gmail.com (S.R.V.); 2 Faculty of Medicine, University of Porto, 4200-319 Porto, Portugal; miguel.neves.silva@ulssjoao.min-saude.pt (M.S.); sandra.rebelo@ulssjoao.min-saude.pt (S.R.); 3 Department of Internal Medicine, Hospital Garcia de Orta, Unidade Local de Saúde de Almada-Seixal, 2805-267 Almada, Portugal; catarina.pestana.santos@ulsas.min-saude.pt; 4 Department of Radiology, Hospital de São João, Unidade Local de Saúde de São João, 4200-319 Porto, Portugal; 5 Department of Pathology, Hospital de São João, Unidade Local de Saúde de São João, 4200-319 Porto, Portugal; 6 Institute of Health Innovation and Research, University of Porto, 4200-135 Porto, Portugal

**Keywords:** pneumococcal infections, *Streptococcus pneumoniae*, Austrian syndrome, endocarditis, bacterial, meningitis, pneumococcal, aneurysm, infected, aortitis

## Abstract

Background: Invasive pneumococcal disease (IPD) is a severe form of *Streptococcus pneumoniae* infection, defined by its isolation from a normally sterile site (such as blood or cerebrospinal fluid (CSF)), and carries high mortality (approximately 20% in adults, rising to 30–40% in the elderly), particularly with multiorgan involvement. Its rarer forms include endocarditis, aortitis, infected aneurysms and Austrian syndrome (the triad of pneumonia, meningitis and endocarditis). Case Presentation: We describe two cohabitating brothers, unvaccinated against pneumococcus, both smokers and with alcohol use disorder, admitted to the hospital two weeks apart. The first patient, aged 53, presented with pneumonia, meningitis/ventriculitis and aortitis progressing to infected aortic aneurysms, requiring two operations and prolonged antibiotic therapy. *S. pneumoniae* was isolated from blood cultures and CSF *S. pneumoniae* real-time polymerase chain reaction (rt-PCR) was positive. The second patient, aged 48, presented with pneumonia, meningitis and aortic valve endocarditis (Austrian syndrome), requiring valve replacement. CSF was also rt-PCR positive for *S. pneumoniae* and blood cultures grew *S. pneumoniae.* An immunodeficiency work-up was normal in both brothers. Conclusions: These cases illustrate the rare, near-simultaneous occurrence of severe IPD in cohabiting siblings and the complexity of its cardiovascular and neurological complications, underlining the importance of early diagnosis, a multidisciplinary approach and pneumococcal vaccination of at-risk groups; when severe disease occurs without classic risk factors, an underlying immunodeficiency should be excluded.

## 1. Introduction

*Streptococcus pneumoniae* is a leading cause of community-acquired pneumonia [1] and can also cause invasive pneumococcal disease (IPD), a more severe presentation defined by the isolation of *S. pneumoniae* from a normally sterile site [1,2], such as in bacteraemia, meningitis, or endocarditis. *S. pneumoniae* colonises the upper respiratory tract of up to 50–80% of children and 2–10% of adults [3,4]; transition from colonisation towards invasive disease depends on bacterial virulence factors and host immunity [5]. Recognised risk factors for IPD include extremes of age (<2 and ≥65 years); immunosuppressive conditions (transplant, HIV, asplenia, haematological malignancies); chronic diseases (pulmonary, cardiac, renal, hepatic, diabetes); smoking; alcohol use disorder; prior influenza infection; and socioeconomic factors such as poverty and overcrowding [6].

Pneumococcal infections are an important cause of morbidity and mortality worldwide, particularly in their invasive forms [2]. IPD carries a high case-fatality rate, estimated at approximately 20% overall in adults and rising to 30–40% in the elderly, with mortality further increased by septic shock, multiorgan involvement and underlying comorbidities [1,2].

The most common presentations of IPD are bacteraemic pneumonia, bacteraemia without an identifiable focus, and meningitis [7]. Rarer but particularly severe presentations include infective endocarditis (IE), vascular involvement such as aortitis and infected aneurysms, and purpura fulminans [8].

Pneumococcal endocarditis is a rare but aggressive form of IE, accounting for fewer than 2% of all IE cases [9]. It predominantly affects native valves—particularly the aortic valve—, typically in patients with predisposing conditions for IPD but often without prior valvular heart disease [9]. Although uncommon in the antibiotic and vaccine era, it still carries a high mortality, with heart failure and shock developing more frequently than in non-pneumococcal IE and often necessitating early cardiac surgery [9]. It has been reported to coexist with pneumonia and meningitis in 16–26.1% of cases [9,10]. Infected aortic aneurysms account for fewer than 2% of all aortic aneurysms and carry a mortality of up to 40%, with *S. pneumoniae* only rarely implicated [11].

Austrian syndrome—the triad of pneumococcal pneumonia, meningitis and endocarditis—is a severe form of IPD, first recognised by William Osler in 1881 and later attributed to *S. pneumoniae* by Robert Austrian in 1957 [12]. The syndrome is rare, but its exact incidence remains unknown; a 2024 systematic review identified 71 cases from 69 case reports published between 1991 and 2022 [12]. It predominantly affects middle-aged men (mean age 56.5 years; male-to-female ratio 2.4:1), with alcohol use disorder being the most commonly reported risk factor (present in approximately 41% of cases) [12]. The aortic valve is most frequently involved, and the syndrome carries a high case-fatality rate of approximately 28%, with most patients requiring intensive care and prolonged antibiotic therapy, often combined with valvular surgery [12].

## 2. Case Presentation

The first patient was a 53-year-old man with a history of smoking, alcohol use disorder, and mild cognitive impairment; imaging also revealed features of chronic liver disease. The patient had not received pneumococcal vaccination. He presented to the emergency department (ED) with a two-week history of myalgia, cough, headache, abdominal pain, and vomiting. On examination, he was febrile with diffuse abdominal tenderness and no other findings. Laboratory tests showed raised inflammatory markers, mildly elevated markers of hepatic cytolysis and cholestasis, and severe hyponatraemia (Table 1). Vitamin D deficiency was also documented (11 ng/mL). *S. pneumoniae* urinary antigen was negative. A chest radiograph showed no consolidation, and abdominopelvic computed tomography (CT) angiography revealed non-specific infrarenal aortitis. Sepsis of unknown origin was presumed, and he started taking piperacillin/tazobactam 4.5 g every 8 h and vancomycin 1000 mg every 12 h (following the loading dose).

**Figure 1:**
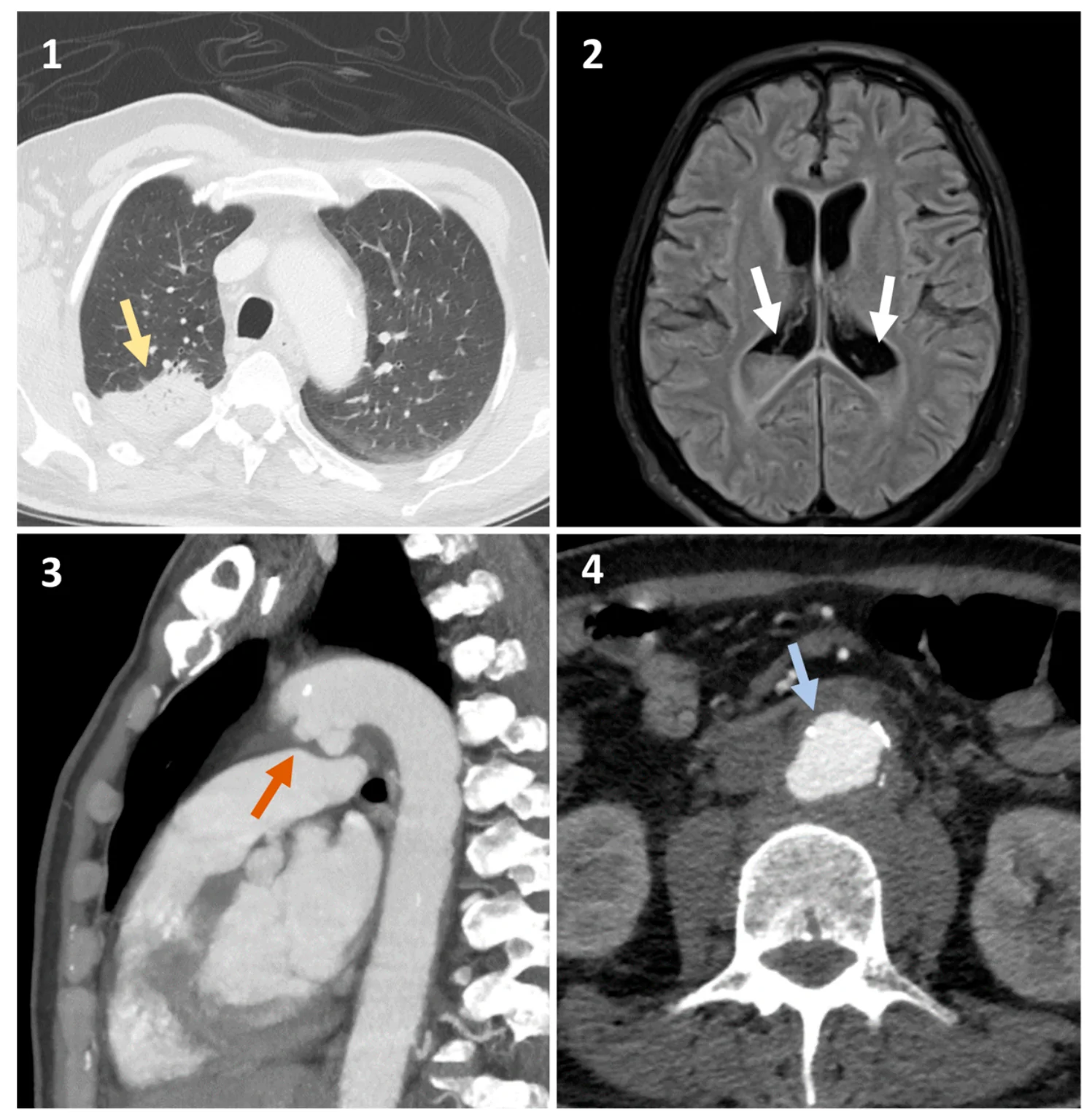
Imaging findings in Patient 1: (**1**) chest CT showing right upper lobe pneumonia (yellow arrow); (**2**) brain magnetic resonance imaging (MRI) showing ventriculitis (white arrows); (**3**) abdominopelvic CT showing aortic arch aneurysm, maximum diameter 35 mm (red arrow); (**4**) abdominopelvic CT showing infrarenal aortic aneurysm, maximum diameter 52 mm (blue arrow).

During admission, he remained febrile and developed a depressed level of consciousness. Cranial CT was suggestive of ventriculitis (Figure 1), and lumbar puncture (LP) yielded turbid CSF with neutrophilic pleocytosis, elevated protein, and undetectable glucose (Table 1). Penicillin-susceptible (MIC = 0.012 mg/L) *S. pneumoniae* (serotype not determined) was isolated from blood cultures obtained in the ED, and CSF *S. pneumoniae* real-time polymerase chain reaction (rt-PCR) was positive, confirming invasive pneumococcal disease with central nervous system (CNS) involvement.

Piperacillin–tazobactam was discontinued, and ceftriaxone 2 g every 12 h and ampicillin 2 g every 4 h were initiated, while vancomycin was continued. Adjunctive corticosteroids were not administered. Following isolation of *S. pneumoniae,* vancomycin and ampicillin were discontinued after four and two days of administration, respectively, while ceftriaxone was continued for a further four days, with subsequent de-escalation to intravenous penicillin at 24 million units/day based on susceptibility results.

Transthoracic echocardiography (TTE) showed no evidence of infective endocarditis but raised suspicion of aortic arch aortitis. Repeat thoraco-abdominopelvic CT angiography demonstrated right upper lobe consolidation and aortic wall thickening at the arch-to-descending-aorta transition and in the infrarenal segment, with parietal ulceration, mural thrombi, and surrounding fat stranding. Surgery was not initially indicated. With worsening abdominal pain, follow-up imaging showed progression to infected aneurysms at both sites (Figure 1). Given the clinical and radiological deterioration, a two-stage surgical approach was undertaken, comprising vascular and cardiothoracic surgery—an aorto-biiliac bypass with graft placement, followed by replacement of the distal aortic arch and proximal descending thoracic aorta with a conduit.

He completed six weeks of intravenous penicillin after the final surgery, followed by suppressive therapy with oral amoxicillin. Antibiotic therapy was discontinued after a follow-up positron emission tomography (PET) scan showed no evidence of residual infection, for a total treatment duration of approximately 33 months.

The second patient, a sibling and household contact of the first, was a 48-year-old man with a history of smoking, chronic bronchitis, and alcohol use disorder, and no pneumococcal vaccination. He presented to the ED two weeks after his brother, with a one-week history of fever, prostration, disorientation, and confused speech. On admission, he was febrile and confused, with barely intelligible, inappropriate speech and no other relevant findings. Investigations were notable for raised inflammatory markers, mildly elevated markers of hepatic cytolysis and cholestasis, and moderate hyponatraemia (Table 2). The patient also had vitamin D deficiency (8 ng/mL). *S. pneumoniae* urinary antigen was positive. A chest radiograph showed right lower lobe pneumonia. He was started on ceftriaxone 2 g every 12 h, ampicillin 2 g every 4 h and dexamethasone, and cranial CT revealed hydrocephalus. LP yielded CSF with neutrophilic pleocytosis, elevated protein, and undetectable glucose (Table 2).

While in the ED, he had a generalised tonic–clonic seizure and a fluctuating level of consciousness. He was admitted to the intensive care unit and required orotracheal intubation and vasopressor support.

**Figure 2:**
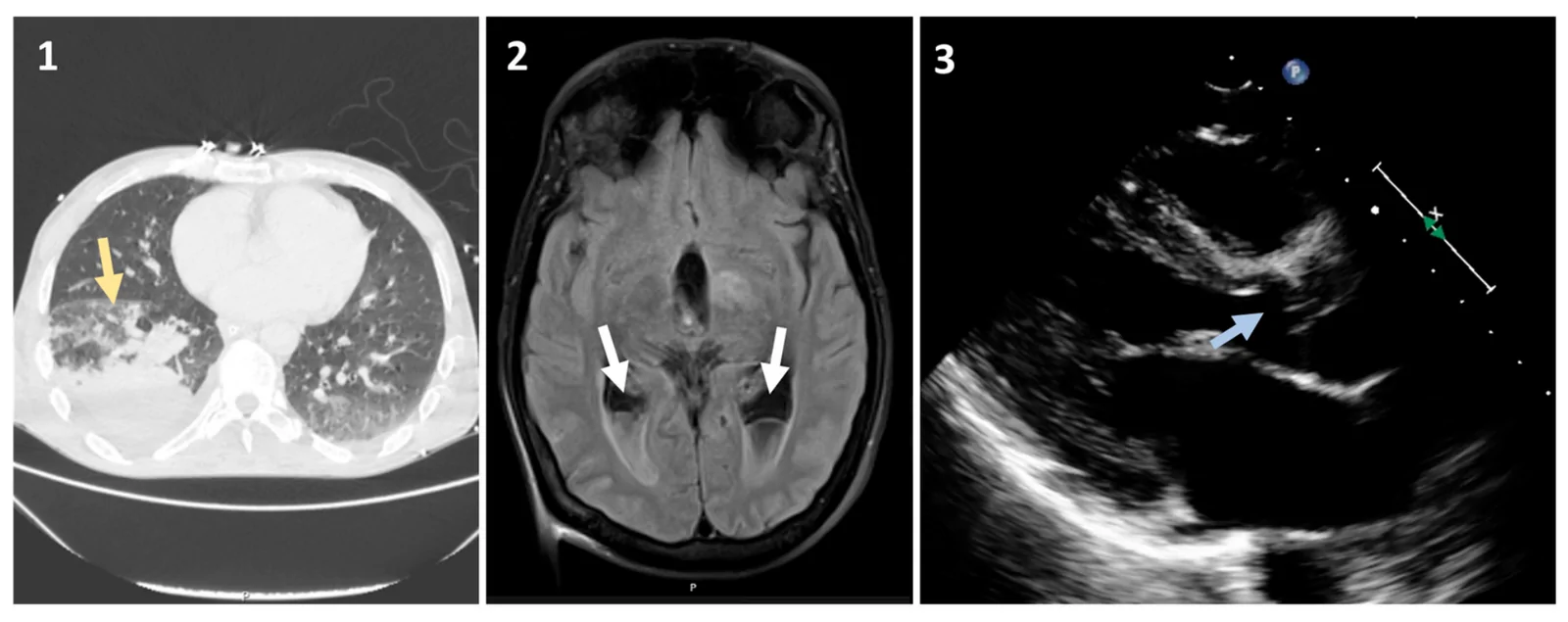
Imaging findings in Patient 2: (**1**) chest CT showing right lower lobe pneumonia (yellow arrow); (**2**) brain magnetic resonance imaging (MRI) showing ventriculitis (white arrows); (**3**) aortic valve vegetation (blue arrow).

Repeat cranial CT showed ventriculitis and worsening hydrocephalus (Figure 2), requiring an external ventricular drain. Subsequent imaging revealed a new left-hemisphere hypodensity consistent with a probable embolic infarct. TTE then demonstrated aortic valve endocarditis (Figure 2) with aortic regurgitation, initially mild but progressing to severe; he underwent aortic valve replacement with a bioprosthesis. Valve culture was negative.

Once CSF was PCR-positive for *S. pneumoniae* serotype 8 and blood cultures grew penicillin-susceptible (oxacillin 1 µg zone diameter ≥ 20 mm) *S. pneumoniae,* therapy was narrowed to ampicillin and continued until two weeks after cardiac surgery. The total duration of antibiotic therapy was 72 days.

After a prolonged admission, he was discharged to a long-term care facility for motor rehabilitation.

HIV testing was negative in both brothers, and an immunological work-up revealed no abnormalities, including serum immunoglobulin levels (IgG, IgA, IgM, IgE), IgG subclasses, complement system assessment (C1, C3, C4, C5) and total haemolytic complement activity (CH50), lymphocyte subset analysis (CD4, CD8, and NK cells), and serum protein electrophoresis. Genetic testing was subsequently performed. In patient 1, a heterozygous variant in the *TNFRSF13B* gene and a hemizygous variant in the *XIAP* gene were identified, while patient 2 was found to carry the same hemizygous *XIAP* variant. The *TNFRSF13B* variant was not considered disease-causing in this context, as patient 1 lacked clinical and immunological features suggestive of common variable immunodeficiency, including hypogammaglobulinemia, impaired vaccine responses, and abnormalities in B-cell subsets. The *XIAP* variant was classified as a variant of uncertain clinical significance, as both patients lacked clinical features compatible with X-linked lymphoproliferative disease.

## 3. Discussion

These cases illustrate severe complications of IPD in two cohabiting brothers admitted two weeks apart. The first developed pneumonia, meningitis, and aortitis requiring two surgeries; the second developed pneumonia, meningitis, and endocarditis (Austrian syndrome) requiring valve replacement.

The occurrence of IPD in two cohabiting siblings within a two-week interval suggests household transmission of *S. pneumoniae*, presumably of the same serotype. Asymptomatic nasopharyngeal carriage is a prerequisite for both transmission and invasive disease, and the household is a well-recognised setting for horizontal pneumococcal spread through close contact [5]; pneumococcal carriage is estimated to be acquired within the household in 29–50% of cases, depending on household size and the age composition of its members [13]. In a large population-based study, household relatives of patients with IPD had an increased risk of invasive pneumococcal infection during the first year following the index case, suggesting that shared environmental risk factors within the household may also increase susceptibility to infection during a common time period [14]. Published reports of intrafamilial transmission of pneumococcal disease are scarce. One case report described three couples with pneumococcal pneumonia caused by isolates of the same serotype [15]. More recently, two cohabiting cousins with pneumococcal spontaneous bacterial peritonitis caused by the same serotype were reported, which was considered as evidence of intrafamilial transmission [16]. Similarly to these reports, our cases involved pneumococcal infection occurring among cohabitating family members within a short time interval. However, the absence of serotyping in the first patient precludes confirmation of this hypothesis.

Both alcohol use disorder and cigarette smoking, present in our patients, are modifiable risk factors for IPD, each operating through overlapping but distinct immunosuppressive mechanisms. Chronic alcohol use impairs pulmonary host defences at multiple levels: it disrupts mucociliary clearance, induces alveolar phagocytic dysfunction, suppresses neutrophil recruitment and promotes aspiration of upper airway flora, collectively resulting in a 2.9- to 11.4-fold increased risk of IPD [17,18]. Cigarette smoking, the strongest independent risk factor for IPD among immunocompetent nonelderly adults (OR 4.1; 95% CI 2.4–7.3), with a clear dose–response relationship and a population attributable risk of 51% [19], similarly compromises innate pulmonary immunity: it impairs complement-mediated phagocytosis, attenuates nasal inflammatory mediator expression and neutrophil recruitment during pneumococcal colonisation, suppresses neutrophil bactericidal activity, inhibits Th17 responses to *S. pneumoniae*, and directly promotes pneumococcal biofilm formation [20]—all of which favour persistent colonisation and subsequent invasion. Together, these two lifestyle exposures create a state of multifactorial functional pulmonary immunosuppression—spanning mechanical barriers, innate cellular immunity, and adaptive mucosal responses—that substantially increases susceptibility to pneumococcal colonisation, invasion, and severe disease [18]. Alcohol use disorder and smoking have also been reported in cases of MRSA pericarditis, another rare but potentially fatal invasive bacterial infection [21].

The first patient also had imaging findings suggestive of chronic liver disease, although there was no biochemical evidence of hepatic dysfunction. An underlying hepatic impairment, if present, could potentially have contributed to impaired clearance of circulating bacteria and complement dysfunction, increasing susceptibility to invasive infections, particularly those caused by encapsulated organisms such as *S. pneumoniae* [22,23]. Finally, both patients had vitamin D deficiency, which may represent an additional factor contributing to increased susceptibility to bacterial infections [24].

Although these identifiable risk factors are recognised to increase susceptibility to IPD, their presence does not exclude a coexisting primary or secondary immunodeficiency—hypogammaglobulinaemia has been found in at least 13.5–31.6% of adult IPD cases, and primary immunodeficiencies were diagnosed in 19% of young adults after a first invasive encapsulated bacterial infection [25,26]. Therefore, immunological evaluation should be strongly considered in patients with IPD, particularly when the presentation is severe, recurrent, or disproportionate to the identified risk factor. In the presented cases, a hemizygous variant in the *XIAP* gene was identified in both patients, while patient 1 also carried a heterozygous variant in the *TNFRSF13B* gene. The *XIAP* variant is not typically associated with IPD; however, *TNFRSF13B* variants have been associated with common variable immunodeficiency and may contribute to increased susceptibility to infections. Despite this association, the *TNFRSF13B* variant was not considered to be disease-causing in patient 1, given the absence of clinical and immunological features of common variable immunodeficiency.

In our patients, IPD evolved to two rare and severe complications. The first patient developed pneumococcal aortitis, which progressed to infected aneurysms involving both the aortic arch and the infrarenal aorta—an exceptionally uncommon manifestation, as *S. pneumoniae* is only rarely implicated in infected aortic aneurysms. Management required a staged vascular and cardiothoracic surgical approach, followed by prolonged antibiotic therapy. The second patient developed aortic valve endocarditis on a previously normal native valve, consistent with the typical pattern of pneumococcal endocarditis. The disease course was complicated by progressive aortic regurgitation and a probable embolic cerebral infarction, ultimately requiring aortic valve replacement. This patient also fulfilled the criteria for Austrian syndrome, presenting the complete classic triad of pneumococcal pneumonia, meningitis and endocarditis. His profile—a middle-aged man with alcohol use disorder and aortic valve involvement requiring surgery—closely matches the syndrome’s typical presentation and illustrates its severity and the frequent need for combined medical and surgical management.

Pneumococcal vaccination of at-risk groups remains a key measure to prevent this infection and its complications [1,2,27]. *S. pneumoniae* serotype 8 has emerged as one of the most prevalent non-PCV13 serotypes causing IPD in the post-conjugate vaccine era, accounting for up to 19–20% of all IPD cases, driven by serotype replacement following PCV7/PCV13 introduction [28,29]. PCV7, PCV13 and PCV15 do not cover serotype 8, while PCV20, PCV21 and PPSV23 do [30].

Serotype 8 is characterised by a high case-to-carrier ratio (high invasiveness)—it is rarely detected in nasopharyngeal carriage, but has a strong propensity to cause invasive disease once colonisation occurs, a feature shared with serotypes 1 and 7 [29,31]. The high invasiveness of serotype 8 is driven by its capsule’s ability to enhance recruitment of complement factor H (via the surface protein PspC), which inhibits C3b/iC3b opsonization and thereby allows evasion of complement-mediated neutrophil phagocytosis; additionally, the dominant CC53/GPSC3 lineage (carrying PspC variant 6.11) demonstrates increased lung cell adhesion, enhanced biofilm formation, and superior phagocytosis evasion compared to other co-circulating lineages [32,33]. Paradoxically, despite this high invasiveness, serotype 8 is associated with a relatively low case-fatality rate and tends to affect younger, healthier individuals (median age 59 years, with lower comorbidity prevalence than PCV13 serotypes) [28].

In Portugal, following the introduction of PCV13 in the National Immunisation Programme in 2015 for routine childhood vaccination at 2, 4 and 12 months, *S. pneumoniae* serotype 8 has emerged as the leading cause of adult IPD. Its proportion increased progressively, from 6.2% of adult IPD cases in 2009–2011 to 11% in 2012–2014 and 19% in 2015–2018—surpassing serotype 3 (15%) in the latter period—and reaching 30% in 2018–2023 [34,35,36,37]. Together, serotypes 8 and 3 have accounted to approximately 40% of all adult IPD cases during the most recent surveillance period (2018–2023) [34,35,36,37]. Despite its predominance in invasive disease, serotype 8 is rarely detected in nasopharyngeal carriage among Portuguese children and tends to cause disease in younger adults compared to PCV13 serotypes, consistent with its characteristically high invasiveness [36,38].

In December 2024, PCV13 was replaced by PCV20 in the Portuguese National Immunisation Programme for both routine childhood vaccination and adults with risk factors [27,39]. Nevertheless, as in most European countries, including the United Kingdom, pneumococcal vaccination in Portugal is recommended only for adults aged ≥65 years or those with specific chronic or immunocompromising conditions; smoking and alcohol use disorder are not considered independent indications for vaccination. In contrast, current United States recommendations recognise both smoking and alcohol use disorder as standalone indications for pneumococcal vaccination in adults aged ≥19 years [40]. Under current Portuguese recommendations, the first patient was eligible for vaccination due to chronic liver disease, whereas the second qualified due to chronic bronchitis [27]. However, these conditions may not be recognised until later in the disease course, meaning that patients with smoking or alcohol use disorder may already be at increased risk of IPD before becoming eligible for vaccination. These observations support the consideration of smoking and alcohol use disorder as independent indications for pneumococcal vaccination in adults, as already recommended in the United States, rather than relying on the later emergence of a qualifying comorbidity.

## 4. Conclusions

These two cases are remarkable for the rare, near-simultaneous occurrence of severe IPD in cohabiting siblings—strongly suggesting household transmission—and for the unusual concurrence of life-threatening cardiovascular complications (infected aortic aneurysms in one brother and Austrian syndrome with aortic valve endocarditis in the other) alongside severe neurological involvement (meningitis, ventriculitis, hydrocephalus, seizures and embolic infarction). They illustrate the potential for IPD to evolve into complex multisystem disease requiring early recognition and coordinated multidisciplinary medical and surgical management. These cases also reinforce the pivotal role of pneumococcal vaccination in preventing IPD and its complications among at-risk populations: although neither smoking nor alcohol use disorder currently constitute a standalone indication for vaccination under Portuguese or most European recommendations, both are well-established modifiable risk factors for IPD; therefore, extending vaccination recommendations to include such populations should be considered. Finally, severe IPD occurring in the absence of classic risk factors—or with clinical severity disproportionate to the identified risk factors—should prompt investigation for an underlying immunodeficiency.

## Figures and Tables

**Table 1:** Blood and cerebrospinal fluid (CSF) laboratory results for Patient 1.

Parameter (Blood)	Value
Haemoglobin	9.2 g/dL
Leucocytes	24.17 × 10^9^/L
Neutrophils	22.72 × 10^9^/L (94%)
Platelets	512 × 10^9^/L
Creatinine	0.91 mg/dL
Urea	46 mg/dL
Sodium	120 mEq/L
Potassium	4.3 mEq/L
Chloride	88 mEq/L
AST	76 U/L
ALT	78 U/L
GGT	187 U/L
Alkaline phosphatase	217 U/L
Total bilirubin	0.95 mg/dL
C-reactive protein	312.7 mg/L
Parameter (CSF)	Value
Total cells	8651/µL
Polymorphonuclear cells	6748/µL (78.3%)
Mononuclear cells	1871/µL (21.7%)
Red blood cells	1800/µL
Total protein	6.05 g/L
Glucose	<10 mg/dL

AST—aspartate aminotransferase; ALT—alanine aminotransferase; CSF—cerebrospinal fluid; GGT—gamma-glutamyl transferase.

**Table 2:** Blood and cerebrospinal fluid (CSF) laboratory results for Patient 2.

Parameter (Blood)	Value
Haemoglobin	11.3 g/dL
Leucocytes	21.81 × 10^9^/L
Neutrophils	19.00 × 10^9^/L (87%)
Platelets	599 × 10^9^/L
Creatinine	0.62 mg/dL
Urea	24 mg/dL
Sodium	126 mEq/L
Potassium	2.8 mEq/L
Chloride	87 mEq/L
AST	98 U/L
ALT	118 U/L
GGT	63 U/L
Alkaline phosphatase	110 U/L
Total bilirubin	1.13 mg/dL
C-reactive protein	218.7 mg/L
Parameter (CSF)	Value
Total cells	302/µL
Polymorphonuclear cells	270/µL (89.4%)
Mononuclear cells	32/µL (10.6%)
Red blood cells	200/µL
Total protein	7.93 g/L
Glucose	<10 mg/dL

AST—aspartate aminotransferase; ALT—alanine aminotransferase; CSF—cerebrospinal fluid; GGT—gamma-glutamyl transferase.

## Data Availability

The original contributions presented in this study are included in the article. Further inquiries can be directed to the corresponding author.

## Abbreviations

The following abbreviations are used in this manuscript:

CNS	Central Nervous System
CT	Computed Tomography
CSF	Cerebrospinal Fluid
ED	Emergency Department
HIV	Human Immunodeficiency Virus
IE	Infective Endocarditis
IPD	Invasive Pneumococcal Disease
MRI	Magnetic Resonance Imaging
LP	Lumbar Puncture
PET	Positron Emission Tomography
PCV	Pneumococcal Conjugate Vaccine
rt-PCR	Real-time Polymerase Chain Reaction
TTE	Transthoracic Echocardiography
